# A population-based analysis of leaving the hospital against medical advice: incidence and associated variables

**DOI:** 10.1186/1472-6963-13-415

**Published:** 2013-10-14

**Authors:** Allen Kraut, Randy Fransoo, Kendiss Olafson, Clare D Ramsey, Marina Yogendran, Allan Garland

**Affiliations:** 1 Department of Internal Medicine, University of Manitoba Winnipeg, Manitoba, Canada; 2 Department of Community Health Sciences, University of Manitoba Winnipeg, Manitoba, Canada; 3 Manitoba Centre for Health Policy, University of Manitoba Winnipeg, Manitoba, Canada

**Keywords:** Against medical advice, Epidemiology, Canada, Population based

## Abstract

**Background:**

Prior studies of patients leaving hospital against medical advice (AMA) have been limited by not being population-based or assessing only one type of patient.

**Methods:**

We used administrative data at the Manitoba Centre for Health Policy to evaluate all adult residents of Manitoba, Canada discharged alive from acute care hospitals between April 1, 1990 and February 28, 2009. We identified the rate of leaving AMA, and used multivariable logistic regression to identify socio-demographic and diagnostic variables associated with leaving AMA.

**Results:**

Of 1 916 104 live hospital discharges, 21 417 (1.11%) ended with the patient leaving AMA. The cohort contained 610 187 individuals, of whom 12 588 (2.06%) left AMA once and another 2 986 (0.49%) left AMA more than once. The proportion of AMA discharges did not change over time. Alcohol and drug abuse was the diagnostic group with the highest proportion of AMA discharges, at 11.71%. Having left AMA previously had the strongest association with leaving AMA (odds ratio 170, 95% confidence interval 156–185). Leaving AMA was more common among men, those with lower average household incomes, histories of alcohol or drug abuse or HIV/AIDS. Major surgical procedures were associated with a much lower chance of leaving the hospital AMA.

**Conclusions:**

The rate of leaving hospital AMA did not systematically change over time, but did vary based on patient and illness characteristics. Having left AMA in the past was highly predictive of subsequent AMA events.

## Background

Between 1 – 2% of hospitalized patients [1-4] leave hospital against medical advice (AMA). The proportion has varied with study location and diagnosis, ranging from 0.6% in rural hospitals [5] to 13% in inner city hospitals [6], and from 0.1% among postpartum patients [7] to 51% for people in an anorexia nervosa inpatient treatment program [8]. The variable having the strongest reported association with leaving AMA is having done so before [5,6,9,10]. Other variables associated with leaving AMA have been younger age, male sex, membership in a visible minority, lower socioeconomic status, absence of health insurance, substance abuse, psychiatric disorders, and urban residency [1-7,9-17].

We have recently shown using a large population based dataset, that leaving AMA is associated with increased hospital readmissions and mortality at all times up to 180 days post discharge [18] building on limited previous work [4,9,14,19]. As there is little understanding of what is responsible for these adverse outcomes, identification of patients at high risk of leaving AMA is important in determining the cause of these poor outcomes and in the design of interventions to try and offset them.

The major limitations of the existing studies on leaving AMA are that most were based on localized experiences with limited numbers and follow-up, [5,6,10,14,20] were limited to one type of admission diagnosis,[4,7,9,12,13,16,17,19],[20] or were not population based [1,3]. Many studies also did not adjust for confounding variables. The objective of this research was to address these limitations by utilizing a large, population-based data system containing comprehensive information about patients, their diseases, and their hospitalizations to identify variables independently associated with leaving hospital AMA.

## Methods

We used administrative hospital abstract data collected by the Department of Health of the Canadian province of Manitoba, housed in de-identified form at the Manitoba Centre for Health Policy (MCHP). Manitoba has a population of 1.2 million; its two urban areas contain 61% of the population. The government-funded health care system covers all provincial residents. The administrative data contains comprehensive health-related information, and have been linked to other data including census-based socioeconomic information, and vital status. MCHP data has been used and validated extensively to study a wide range of medical outcomes [21,22].

All Manitoba residents ≥18 years of age, discharged alive after admission to provincial acute care hospitals from April 1, 1990 to February 28, 2009 were identified. Because a patient can undergo inter-hospital transfer within a single episode of hospital care, we identified two abstracts as representing a transfer and therefore part of a single hospital episode if: (i) hospital entry of the later abstract was within one calendar day of the previous hospital separation, and (ii) an acute care hospital was the “discharge to” location of the earlier abstract, and/or the “admitted from” location of the later abstract. The exception was that two hospital abstracts were considered as separate hospital episodes if the earlier one indicated that the patient left AMA. Same day surgeries were not considered admissions in this dataset.

In Manitoba, hospital abstracts are collected in each hospital by centrally trained data abstractors using uniform definitions, data collection methods, and data entry software. A specific discharge code for AMA is used. Episodes of care were designated as AMA or non-AMA depending on the presence of this code in the last hospital abstract of each hospital episode.

Age, sex, hospital, fiscal year of admission, and postal code of residence were obtained from the first hospital abstract for each episode of hospital care. Fiscal years run from April 1st to the following March 31st and will be referred to by the calendar year of the start of the period. Length of stay was calculated from the admission and discharge dates and times of the hospital episode. Whether the individual had a prior AMA discharge during the five years before the current admission was also identified.

Socioeconomic status (SES) was based on average household income within geographic dissemination areas, based on the 2001 Canadian census; in Manitoba these area-level census tracts contain an average of 550 persons. These were separately divided into quintiles for rural and urban residents, with 1 being the lowest and 5 the highest income quintile. People living in areas where the Canadian census does not calculate an average household income formed an eleventh SES category called “not calculated” (NC). Most people in the NC category are residents in nursing homes, other chronic care facilities, or penitentiaries.

Diagnosis was derived from the “Most Responsible Hospital Diagnosis” [23] i.e. the diagnosis responsible for the majority of the hospital stay, obtained from the final hospital abstract of each hospital episode. Diagnoses were initially grouped into the 18 main ICD-9-CM chapter headings [24]. Headings with low counts were collapsed, and specific diagnostic entities of interest were extracted from major headings; for a total of 23 diagnoses or diagnostic categories. The only discharges that were excluded were the small number of hospital episodes that lacked discharge diagnoses.

Whether the hospital episode included a major surgical procedure was identified. From 2004 onwards, this information was based on Canadian reporting standards for hospital abstracts, the Case Mix Group (CMG) system. Before 2004, it was based on the similar Diagnosis Related Groups system [25,26].

Hospitals were categorized into: the three urban tertiary hospitals in Manitoba, the four urban community hospitals grouped together, and the rural hospitals grouped together. To assess for changes over time, years were groups as: 1990–1993, 1994–1998, 1999–2003, and 2004–2008.

For co-morbidities, the 31 conditions described by Elixhauser et al. [27] were identified from all diagnoses in the hospital discharge abstracts, using the coding described by Quan *et al*. [28]. For this purpose we included the index hospitalization and all hospital diagnoses for all hospitalizations within one year backwards in time [29,30]. Although the 31 conditions separately codified diabetes with and without chronic complications, in our data these two were not accurately distinguished prior to 2006, so the two subcategories were collapsed together.

We performed external validation of the AMA designation in the administrative data, using 291 hospital abstracts where true AMA status was identified by reading the final nurse and physician progress notes in the hospital charts. These charts were chosen in an approximate 1:2 ratio of AMA:nonAMA as indicated by independently acquired data used by our Department of Medicine. All 198 patients who did not leave AMA were correctly identified as such in the abstracts (specificity 100%, 95% C.I., 98.2-100%). However, only 81 of 93 patients who left AMA were correctly coded in the abstracts as having done so (sensitivity 87%, 95% C.I., 78.5-93.2%). In such a cohort, the indication of AMA status in the hospital abstracts would have a positive predictive value of 100%, and a negative predictive value of 99.86%.

To identify independent factors independently associated with patients leaving the hospital AMA, we constructed multivariable logistic regression models. Independent variables were the hospital diagnosis, co-morbidities, sex, age, hospital type, time period, SES, and whether a major surgical procedure was performed. Anticipating that having left AMA before would have such a strong association with subsequently going AMA that it might confound analysis of other factors, two multivariable regression models are presented -- one including and the other excluding an independent variable representing whether patients had any prior AMA episodes.

Though the unit of measure for this analysis was individual episodes of hospital care, these are not all independent since many individuals had multiple episodes. To account for this clustering of data, we used General Estimating Equations (GEE) [31], with an exchangeable correlation structure and robust (empirical) standard errors. We assessed for multicollinearity among the independent variables using the variance inflation factor, with values under 4 considered acceptable [32]. We report parameter estimates from these models as odds ratios (OR) with 95% confidence intervals (95% CI). We compared regression models using the QIC parameter for GEE models, where lower values indicate a better fit [33].

An important issue in dealing with clustered data is that independent variables may have different between-person and within-person associations with the outcome. We allowed for this in the regressions by considering two separate versions of independent variables [34,35]. For example, the coefficient of the within-person age variable indicates how the probability of leaving AMA varied with age for a *given* person; the coefficient of the between-person age variable indicates the difference in probability of leaving AMA between different people of different ages, each of whom had a single hospitalization. For most of the independent variables only the between-individual version of the variable was included. However, both versions were included for age, and whether the hospitalization included a major surgical procedure.

Univariate comparisons were done using Chi-square tests and t-tests, as appropriate. All analyses were done using SAS version 9.1 (SAS Institute, Cary, NC). A p-value of 0.05 was considered significant.

This proposal was approved by the Research Ethics Board of the University of Manitoba and the Health Information Privacy Committee of the Manitoba Government.

## Results

We identified 1 916 104 hospital episodes in which patients were discharged alive during the 19 year study period, of which 21 417 (1.11%) ended with the patient leaving hospital AMA. This cohort contained 610 187 individuals of whom 12 588 (2.06%) left AMA once, and 2986 (0.49%) left AMA more than once. Thus, 19.1% of people who ever left hospital AMA did so more than once, accounting for 41.2% of all AMA events. The largest number of AMA episodes for one individual was 39. Median length of stay was five days (interquartile range (IQR) 3–9 days) for non-AMA hospital episodes and three days for AMA episodes (IQR 2–6 days) (p < 0.001).

**Figure 1:**
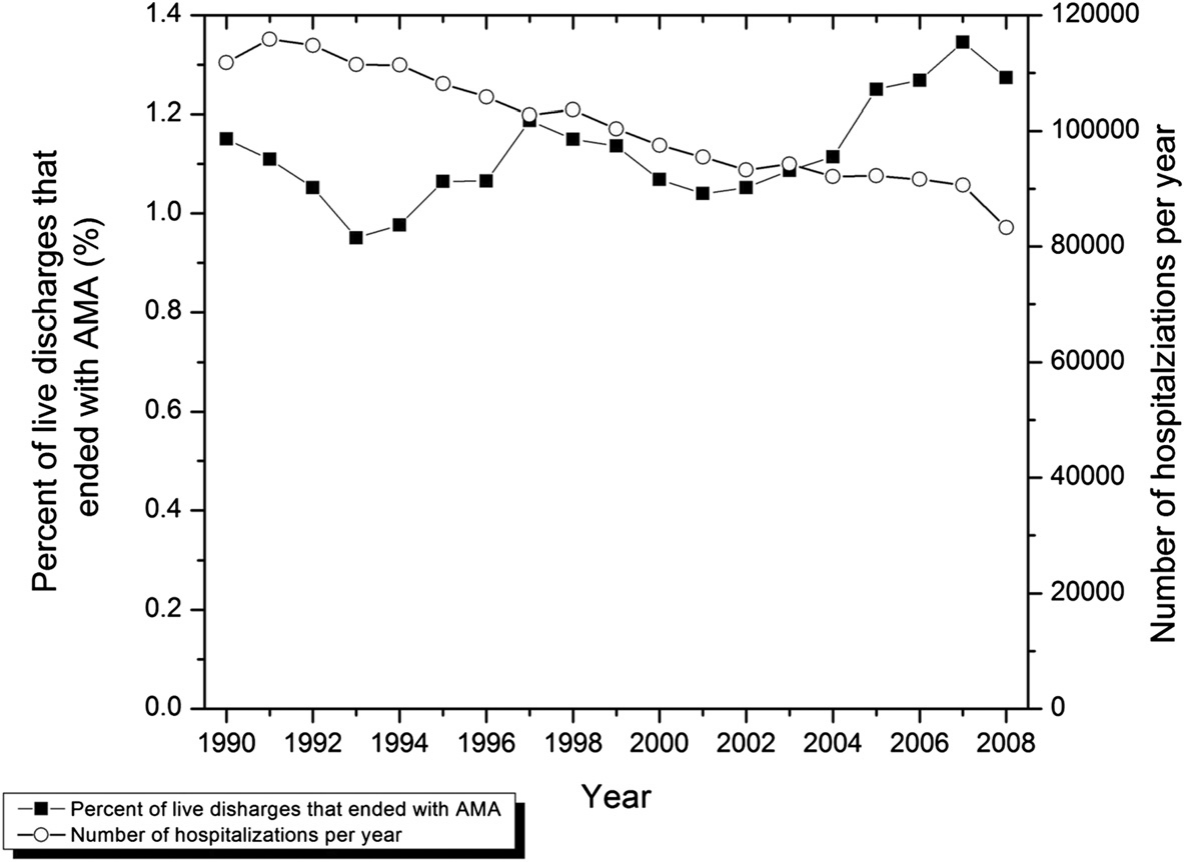
**Total discharges and percentage AMA discharges in Manitoba, Canada.** April 1, 1990 to February 28, 2009.

There was a marked decline in yearly hospital episodes over time, while the percentage of episodes ending with patients leaving AMA fluctuated in an undulating pattern (Figure 1). The slight decline in total hospital episodes observed in the final study year is due to the fact that this period only included 11 months.

Table 1 compares the proportion of hospital episodes ending in an AMA discharge by patient and illness characteristics. Men, and individuals under age 50, had higher proportions of AMA discharges. The association of SES was different for urban and rural residents with a stronger gradient of increasing AMA discharges with lower SES being observed for urban residents. The AMA discharge proportion was also higher for rural hospitals, and for one of the tertiary care hospitals. Individuals who had major surgical procedures were less likely to leave AMA.

**Table 1:** Proportion of live hospital discharges that left against medical advice (AMA), Manitoba, Canada; 1990–2009

***Parameter***	***Percentage AMA discharges***	***Number of AMA discharges***
All episodes	1.11	21 417
Sex		
Male	1.50	10 789
Female	0.89	10 628
Age		
18–34	1.61	8654
35–49	1.96	6136
50–64	1.06	3478
65+	0.42	3149
Income quintile		
Urban 1st quintile (lowest)	2.22	5831
Urban 2nd quintile	1.10	2250
Urban 3rd quintile	0.83	1557
Urban 4th quintile	0.60	913
Urban 5th quintile (highest)	0.45	598
Rural 1st quintile (lowest)	1.67	3864
Rural 2nd quintile	1.00	2147
Rural 3rd quintile	0.78	1534
Rural 4th quintile	0.86	1393
Rural 5th quintile (highest)	0.85	1058
Not calculated*	0.57	272
Admitting Hospital		
Urban tertiary care hospital 1	1.70	5680
Urban tertiary care hospital 2	0.65	1939
Urban tertiary care hospital 3	0.92	1030
Urban community hospitals	0.82	4030
Rural hospitals	1.28	8738
Major surgical procedure group.		
Non-surgical	1.44	19 367
Surgical	0.36	2050

*Includes postal codes for which average household income was not calculated, primarily including institutions such as nursing homes, other chronic care facilities and prisons.

The hospital diagnosis grouping with the highest percentage of AMA discharges was alcohol and drug abuse, at 11.71% (Table 2). Poisoning had an AMA discharge proportion of 7.54%. Alcohol and drug abuse accounted for 13.54% of all of the AMA discharges. Complications of childbirth and the puerperium, although having a relatively low AMA discharge proportion of 0.63%, contributed 10.4% of the total number of AMA discharges due to the high number of admissions for this diagnostic grouping.

**Table 2:** Most responsible hospital diagnosis associated with leaving against medical advice (AMA) discharges, Manitoba, Canada; 1990–2009

***Most responsible hospital diagnosis***	***Total number of episodes***	***AMA episode, (% of live discharges)***	***% of total AMA episodes***
Alcohol or drug abuse	24 768	2900 (11.71)	13.54
Poisonings by pharmaceuticals or alcohol	9684	730 (7.54)	3.41
Alcohol-related liver disease	2202	162 (7.36)	0.76
Diabetic ketoacidosis	4728	267 (5.65)	1.25
Tuberculosis	1353	69 (5.10)	0.32
Intracranial injuries without skull fracture	4759	216 (4.54)	1.01
Injuries with open wounds	10 636	443 (4.17)	2.07
Mental disorders except alcohol and drug abuse	82 349	1745 (2.12)	8.15
Skin and subcutaneous tissue disorders	26 340	522 (1.98)	2.44
Symptoms, signs and ill-defined conditions	102 434	1595 (1.56)	7.45
Endocrine, metabolic, nutritional, immune disorders except diabetic ketoacidosis	40 787	633 (1.55)	2.96
Infectious diseases except tuberculosis	17 965	242 (1.35)	1.13
Trauma, injury and poisoning*	117 929	1473 (1.25)	6.88
Respiratory disorders	138 875	1445 (1.04)	6.75
Hematologic disorders	13 591	129 (0.95)	0.60
Digestive disorders except alcohol-related liver conditions	215 693	2034 (0.94)	9.50
Neurologic disorders	41 637	332 (0.80)	1.55
Circulatory disorders	251 132	1921 (0.76)	8.97
Musculoskeletal system disorders	91 921	607 (0.66)	2.83
Complications of pregnancy, childbirth and the puerperium	355 735	2227 (0.63)	10.40
Genitourinary disorders	115 034	697 (0.61)	3.25
All other diagnoses and categories	127 860	670 (0.52)	3.15
Neoplasms	118 692	358 (0.30)	1.67
Totals:	1 916 104	21 417 (1.11)	100.0

*Except for intracranial injuries without skull fracture, injuries with open wounds, and poisonings by pharmaceuticals or alcohol.

Of the two multivariable GEE regression models for leaving AMA (Table 3), the one including a past history of leaving AMA provided a better fit compared to the model excluding that variable (QIC 169829.0 vs. 200911.2). This reflects that having had any prior AMA events had by far the strongest association with future AMA events (OR 170, 95% CI 156–185). Indeed, its inclusion led to a diminished influence of numerous other variables; for example, the co-morbidity HIV/AIDS was not associated with leaving AMA when the prior AMA variable was included in the model (OR 0.93, 95% CI 0.52-1.67), but was strongly associated when the prior AMA variable was not included (OR 2.85, 95% CI 2.04-4.04). Since including the prior AMA variable masks the association with other relevant characteristics, and because of issues with interpreting the prior AMA variable for patients without any past hospitalizations, for identifying other variables associated with leaving AMA we emphasize the model excluding the prior AMA variable (Table 3, left half).

**Table 3:** Results of logistic regression models for leaving hospital against medical advice (AMA)

	***Excluding prior AMA variable***		***Including prior AMA variable***	
	***Odds ratio***	***95% CI***	***Odds ratio***	***95% CI***
**Between-individual parameters**				
Age (per decade)	0.73*	0.72, 0.74	0.82*	0.80, 0.83
Male sex	1.21*	1.16, 1.26	1.48*	1.41, 1.55
Prior AMA event	--	--	170.0*	156.2, 185.1
*Socioeconomic status (5th [highest] urban quintile as reference group)*				
Urban 1st quintile (lowest)	3.58*	3.21, 3.99	2.54*	2.28, 2.83
Urban 2nd quintile	2.07*	1.84, 2.33	1.78*	1.58, 2.00
Urban 3rd quintile	1.64*	1.45, 1.86	1.41*	1.24, 1.59
Urban 4th quintile	1.20†	1.05, 1.38	1.13	0.99, 1.30
Rural 1st quintile (lowest)	2.48*	2.19, 2.79	1.88*	1.66, 2.13
Rural 2nd quintile	1.49*	1.31, 1.69	1.33*	1.16, 1.52
Rural 3rd quintile	1.30*	1.14, 1.49	1.20‡	1.05, 1.38
Rural 4th quintile	1.39*	1.22, 1.59	1.20‡	1.04, 1.38
Rural 5th quintile (highest)	1.33*	1.16, 1.52	1.29†	1.13, 1.47
Not calculated §	1.49*	1.25, 1.79	1.61*	1.33, 1.94
*Hospital (urban community hospitals as reference group)*				
Urban tertiary hospital 1	1.18*	1.10, 1.26	1.07	1.00, 1.15
Urban tertiary hospital 2	0.87†	0.80, 0.94	0.85†	0.78, 0.92
Urban tertiary hospital 3	0.84†	0.76, 0.93	0.76*	0.68, 0.85
Rural hospitals	1.25*	1.16, 1.35	0.96	0.88, 1.05
*Chronic co-morbid conditions*				
Alcohol abuse	3.82*	3.56, 4.11	1.39*	1.24, 1.55
HIV/AIDS	2.87*	2.04, 4.04	0.93	0.52, 1.67
Drug abuse	2.19*	1.99, 2.42	0.85	0.73, 1.00
Weight loss	1.93*	1.40, 2.64	1.70‡	1.12, 2.60
Blood loss anemia	1.68†	1.25, 2.27	1.01	0.67, 1.51
Diabetes	1.52*	1.41, 1.64	0.97	0.88, 1.07
Deficiency anemia	1.38*	1.13, 1.68	0.80	0.60, 1.05
Peripheral vascular disease	1.36†	1.15, 1.61	0.92	0.77, 1.11
Fluid and electrolyte disorders	1.34*	1.18, 1.53	0.64*	0.52, 0.79
Valvular disease	1.29‡	1.05, 1.59	1.01	0.79, 1.29
Liver disease	1.26‡	1.09, 1.45	0.87	0.70, 1.09
Other neurological disorders	1.24*	1.11, 1.37	0.75†	0.63, 0.88
Peptic ulcer disease without bleeding	1.24	0.95, 1.61	0.69	0.46, 1.04
Chronic pulmonary disease	1.23*	1.12, 1.36	0.84‡	0.75, 0.95
Paralysis	1.21‡	0.98, 1.49	0.79	0.59, 1.06
Coagulopathy	1.20	0.94, 1.53	0.62‡	0.43, 0.90
Hypertension, complicated	1.09	0.85, 1.39	0.78	0.58, 1.04
Obesity	1.09	0.92, 1.30	0.99	0.79, 1.25
Congestive heart failure	1.07	0.96, 1.20	0.96	0.85, 1.09
Renal failure	1.06	0.90, 1.26	0.82	0.68, 1.00
Solid tumor without metastasis	1.04	0.85, 1.27	0.81‡	0.67, 0.99
Rheumatoid arthritis/collagen-vascular disorders	1.04	0.86, 1.26	0.87	0.70, 1.07
Metastatic cancer	0.96	0.81, 1.14	0.84‡	0.71, 1.00
Lymphoma	0.91	0.67, 1.23	0.85	0.65, 1.11
Depression	0.89‡	0.80, 0.99	0.52*	0.45, 0.62
Psychoses	0.87‡	0.76, 1.00	0.60*	0.49, 0.72
Pulmonary circulation disorders	0.85	0.62, 1.17	0.74	0.51, 1.07
Arrhythmia	0.81†	0.72, 0.91	0.69*	0.60, 0.78
Hypertension, uncomplicated	0.74*	0.67, 0.81	0.78*	0.70, 0.86
Hypothyroidism	0.70†	0.57, 0.86	0.80‡	0.64, 1.00
*Most responsible hospital diagnosis (cardiovascular disorders as reference group)*				
Poisoning by pharmaceuticals or alcohol	2.72*	2.25, 3.28	4.16*	3.31, 5.23
Intracranial injuries without skull fracture	2.10*	1.69, 2.61	3.34*	2.64, 4.23
Tuberculosis	2.02*	1.46, 2.79	1.39	0.88, 2.20
Alcohol or drug abuse	1.91*	1.65, 2.20	2.69*	2.25, 3.21
Injuries with open wounds	1.91*	1.62, 2.26	1.71*	1.39, 2.12
Diabetic ketoacidosis	1.69†	1.24, 2.30	0.59‡	0.38, 0.92
Diseases of the skin and subcutaneous tissue	1.44*	1.21, 1.72	1.37‡	1.11, 1.68
Symptoms, signs, and ill-defined conditions	1.31†	1.14, 1.50	1.08	0.91, 1.28
Injury and poisoning||	1.14‡	1.01, 1.29	1.27†	1.11, 1.45
Alcohol-related liver disease	1.06	0.72, 1.56	1.92‡	1.02, 3.63
Endocrine, metabolic, nutritional, immune disorders^¶^	1.05	0.85, 1.29	0.86	0.62, 1.19
Mental disorders^**^	0.96	0.82, 1.13	1.22‡	1.01, 1.48
Diseases of the digestive system^††^	0.86‡	0.76, 0.97	0.62*	0.54, 0.72
Diseases of the musculoskeletal system & connective tissue	0.84‡	0.71, 1.00	0.67*	0.56, 0.80
Diseases of the respiratory system	0.83‡	0.72, 0.96	0.82‡	0.69, 0.96
Infectious and parasitic diseases except tuberculosis	0.61†	0.46, 0.79	0.79	0.58, 1.08
Diseases of nervous system and sense organs	0.59*	0.47, 0.73	0.58*	0.45, 0.74
Diseases of blood and blood-forming organs	0.58†	0.38, 0.87	0.62‡	0.39, 0.99
Diseases of the genitourinary system	0.56*	0.47, 0.65	0.45*	0.38, 0.54
Neoplasms	0.52*	0.42, 0.64	0.61*	0.50, 0.75
Factors influencing health status, congenital abnormalities, or conditions originating in the perinatal period	0.38*	0.33, 0.45	0.47*	0.40, 0.56
Complications of pregnancy, childbirth, puerperium	0.27*	0.24, 0.30	0.33*	0.29, 0.38
Major surgical procedure	0.30*	0.28, 0.32	0.39*	0.36, 0.43
*Time period (1990–93 as reference group)*				
1994-1998	0.96	0.90, 1.03	0.82*	0.76, 0.89
1999-2003	0.91†	0.86, 0.97	0.91‡	0.84, 0.98
2004-2008	0.99	0.93, 1.05	1.03	0.96, 1.11
**Within-individual parameters**				
Age (per decade)	1.02	0.97, 1.07	1.01	0.96, 1.07
Major surgical procedure	0.32*	0.30, 0.34	0.32*	0.30, 0.34

* p < 0.0001.

† p ≥ 0.0001 and < 0.001.

‡ p ≥ 0.001 and < 0.05.

§ Includes postal codes for which average household income was not calculated, primarily institutions such as nursing homes, other chronic care facilities and prisons.

|| Except for intracranial injuries without skull fracture, injuries with open wounds, and poisonings by pharmaceuticals or alcohol.

¶ Except for diabetic ketoacidosis.

** Except for alcohol or drug abuse.

†† Except for alcohol-related liver disease.

There were no consistent changes in the odds of leaving AMA across the 19 year evaluation period. While older patients were less likely to leave AMA than were younger patients, the odds that a *given* patient would leave AMA did not change as that person aged. Men were more likely to leave AMA. Consistent gradients with SES showed that individuals living in areas with lower average household incomes were more likely to leave AMA. There were some differences between hospitals in the odds of patients leaving AMA, with the highest OR being for the rural hospitals.

A number of chronic conditions were significantly associated with an elevated risk of leaving AMA; most prominent among these were alcohol abuse (OR 3.82; 95% CI 3.56-4.11), HIV/AIDS (OR 2.87; 95% CI 2.04-4.04) and drug abuse (OR 2.19; 95% CI 1.99-2.42). The two co-morbid conditions which conferred the lowest odds for leaving AMA, were hypothyroidism (OR 0.70; 95% CI 0.57-0.86) and uncomplicated hypertension (OR 0.74; 95% CI 0.67-0.81).

The chance of leaving AMA varied substantially with the main hospital diagnosis. Compared to the reference group of cardiovascular disorders, the ORs varied 10-fold, from 0.27 to 2.72. All but three of the 22 diagnoses were significantly different than the reference group. Prominent among those more likely to leave AMA were patients with poisonings or overdoses related to medications or alcohol (OR 2.72; 95% CI 2.25-3.28), intracranial injuries without skull fracture (OR 2.10; 95% CI 1.69-2.61) and tuberculosis (OR 2.02; 95% CI 1.46-2.79). Among those with the lowest chance of leaving AMA were patients admitted with problems relating to pregnancy and childbirth (OR 0.27; 95% CI 0.24-0.32), and cancer (OR 0.52; 95% CI 0.47-0.64). Finally, major surgical procedures were associated with a much lower chance of leaving the hospital AMA; this was true both between different people, and comparing between different hospitalizations for a given person.

## Discussion

Using a large, population-based database including all acute hospital admissions among adults over a 19 year period, we found that just over 1% of hospital episodes discharged alive ended with the patient leaving AMA. This fraction did not change systematically over the study period; with the declining rate of hospitalizations over this 19 year period (Figure 1), this translates to a decline over time in the absolute numbers of AMA events per year. As our data does not include information about patients’ reasons for leaving AMA, it does not allow us to further explore whether there were temporal changes in those reasons.

Approximately 20% of people who left AMA did so multiple times, accounting for over 40% of all AMA events. Studying these individuals may be useful to identify steps that could be taken during hospitalization to limit the chance of them leaving AMA again.

Our modeling showed that having left AMA from a prior hospitalization had by far the strongest association with future AMA events; indeed it masked the effect of numerous other relevant variables. Since characteristics relating to an person’s tendency to leave AMA are for the most part present for all of that his/her hospitalizations, the prior AMA variable itself includes some of those other individual characteristics, in effect “absorbing” their independent influence on the tendency to leave AMA again. Omitting the prior AMA variable from the model eliminates this sort of confounding, and permits clearer identification of other variables associated with leaving AMA.

Those other independent variables associated with leaving AMA included younger age, male sex, lower SES, various comorbid conditions and hospital admission diagnoses, and nonsurgical status. Among admission diagnoses associated with a lower odds of leaving AMA were neoplasia which may be associated with a more significant disease burden, congenital anomalies which may be associated with more significant mental or physical impairments making leaving AMA difficult, and conditions associated with pregnancy which may have typical shorter lengths of stay. Two novel findings that extend beyond prior work were also observed. First, the likelihood that a *given* patient would leave AMA did not change as that person aged. This indicates that whatever predisposes people to leave AMA does not change as they age. The second observation was a much lower rate of leaving AMA following hospitalizations that included a major surgical procedure in both the between- and within-individual analyses. Surgical procedures may have lower AMA discharges as many are elective and have early discharge goals. When a surgical procedure is not involved, an individual may feel that they can manage outside the hospital earlier than their caregivers and choose to leave AMA if they have other characteristics associated with AMA discharges.

Substance abuse and HIV/AIDS were strongly associated with leaving AMA, consistent with previous research [3-5,7,9,10,15,20]. Addictions and other risk-taking behaviors may be related to these associations. Furthermore, individuals with these conditions may not have a primary care physician, which has been associated with leaving AMA [9,12].

In our cohort, one of the tertiary urban hospitals, and the rural hospitals had higher odds ratios for patients leaving AMA, while the other two tertiary urban hospitals had lower ones. This could reflect either hospital factors which influence the likelihood of patients leaving AMA, or systematic differences between patients at the different hospitals that are not captured by the individual-level characteristics included in our analysis. Hospital factors such as size, location, and measures of care, have previously been associated with AMA discharges [1,3,15,16]. Our results differ from those of Ibrahim *et al.*[3] in that we found AMA discharges to be more likely from rural hospitals.

Our study has important strengths. The most notable is that it is a large study that included all adult hospitalizations over a 19 year span in an entire Canadian province. Accordingly, unlike prior studies of leaving AMA, our analysis covered an entire population and included patients with medical, mental health, and surgical problems. We also validated the AMA discharge designation which was used, and adjusted for the potential confounding effect of individuals that have multiple hospitalizations. The main limitations of our study relates to its generalizability; since leaving AMA could be influenced by cultural, religious and other social factors, our results may not apply elsewhere. Although Manitoba has a large Aboriginal population, and prior studies have shown that ethnicity is associated with leaving AMA [2,20], our administrative data does not allow identification of Aboriginal individuals. In addition, we lacked information about the severity of acute illness during hospitalization, admission source, and a variety of social and personal factors such as marital status that could have influenced the AMA decision. Finally, we have no information on physician related variables, which may influence whether some discharges are classified as AMA [36].

## Conclusions

In this study we identified patient and illness factors associated with patients who leave the hospital against medical advice. Identifying patients at risk of leaving AMA is an important step in designing approaches to limiting AMA admissions. Future research is needed in identifying the appropriate ways to deal with patients who leave AMA, to promote high quality care for this population, and to alleviate the potential adverse consequences of this type of discharge [36].

## Competing interests

All authors have also completed the Unified Competing Interest form developed by the International Committee of Medical Journal Editors (ICMJE), available on request from the corresponding author.

## Authors’ contributions

AK conceived of the study. All authors oversaw the studies design, contributed to the interpretation of the data, and redrafted the paper for important intellectual content. All authors have approved the final version of the manuscript.

## Pre-publication history

The pre-publication history for this paper can be accessed here:

http://www.biomedcentral.com/1472-6963/13/415/prepub

## References

[B1] Smith DB Telles JL Discharges against medical advice at regional acute care hospitals Am J Public Health 1991 81 212 215 10.2105/AJPH.81.2.212 1899322 PMC1404966

[B2] Franks P Meldrum S Fiscella K Discharges against medical advice: are race/ethnicity predictors? J Gen Intern Med 2006 21 955 960 10.1007/BF02743144 16918741 PMC1831611

[B3] Ibrahim SA Kwoh CK Krishnan E Factors associated with patients who leave acute-care hospitals against medical advice Am J Public Health 2007 97 2204 2208 10.2105/AJPH.2006.100164 17971552 PMC2089112

[B4] Glasgow JM Vaughn-Sarrazin M Kaboli PJ Leaving against medical advice (AMA): risk of 30-day mortality and hospital readmission J Gen Intern Med 2010 25 926 929 10.1007/s11606-010-1371-4 20425146 PMC2917668

[B5] Seaborn MH Osmun WE Discharges against medical advice: a community hospital’s experience Can J Rural Med 2004 9 148 153 15603687

[B6] Anis AH Sun H Guh DP Palepu A Schechter MT O'Shaughnessy MV Leaving hospital against medical advice among HIV-positive patients CMAJ 2002 167 633 637 12358196 PMC122025

[B7] Fiscella K Meldrum S Franks P Post partum discharge against medical advice: who leaves and does it matter? Matern Child Health J 2007 11 431 436 10.1007/s10995-007-0194-3 17334926

[B8] Woodside DB Carter JC Blackmore E Predictors of premature termination of inpatient treatment for anorexia nervosa Am J Psychiatry 2004 161 2277 2281 10.1176/appi.ajp.161.12.2277 15569900

[B9] Weingart SN Davis RB Phillips RS Patients discharged against medical advice from a general medicine service J Gen Intern Med 1998 13 568 571 10.1046/j.1525-1497.1998.00169.x 9734795 PMC1496999

[B10] Baptist AP Warrier I Arora R Ager J Massanari RM Hospitalized patients with asthma who leave against medical advice: characteristics, reasons, and outcomes J Allergy Clin Immunol 2007 119 924 929 10.1016/j.jaci.2006.11.695 17239431

[B11] Corley MC Link K Men patients who leave a general hospital against medical advice: mortality rate within six months J Stud Alcohol 1981 42 1058 1061 7334805 10.15288/jsa.1981.42.1058

[B12] Jeremiah J O'Sullivan P Stein MD Who leaves against medical advice? J Gen Intern Med 1995 10 403 405 10.1007/BF02599843 7472691

[B13] Saitz R Ghali WA Moskowitz MA Characteristics of patients with pneumonia who are discharged from hospitals against medical advice Am J Med 1999 107 507 509 10.1016/S0002-9343(99)00262-4 10569306

[B14] Hwang SW Li J Gupta R Chien V Martin RE What happens to patients who leave hospital against medical advice? CMAJ 2003 168 417 420 12591781 PMC143546

[B15] Brook M Hilty DM Liu W Hu R Frye MA Discharge against medical advice from inpatient psychiatric treatment: a literature review Psychiatr Serv 2006 57 1192 1198 10.1176/appi.ps.57.8.1192 16870972

[B16] Onukwugha EC Shaya FT Saunders E Weir MR Ethnic disparities, hospital quality, and discharges against medical advice among patients with cardiovascular disease Ethn Dis 2009 19 172 178 19537229

[B17] Tawk R Freels S Mullner R Associations of mental, and medical illnesses with against medical advice discharges: the national hospital discharge survey, 1988–2006 Adm Policy Ment Health 2013 40 124 132 10.1007/s10488-011-0382-8 22057857

[B18] Garland A Ramsey CD Fransoo R Olafson K Chateau D Yogendran M Rates of readmission and death associated with leaving hospital against medical advice: a population-based study CMAJ 2013 185 1207 1214 10.1503/cmaj.130029 23979869 PMC3787167

[B19] Onukwugha E Mullins CD Loh FE Saunders E Shaya FT Weir MR Readmissions after unauthorized discharges in the cardiovascular setting Med Care 2011 49 215 224 10.1097/MLR.0b013e31820192a5 21206297

[B20] Fiscella K Meldrum S Barnett S Hospital discharge against advice after myocardial infarction: deaths and readmissions Am J Med 2007 120 1047 1053 10.1016/j.amjmed.2007.08.024 18060925

[B21] Roos LL Gupta S Soodeen RA Jebamani L Data quality in an information-rich environment: Canada as an example Can J Aging 2005 24 Suppl 1 153 170 16080132 10.1353/cja.2005.0055

[B22] Roos NP Roos LL Brownell M Fuller EL Enhancing policymakers’ understanding of disparities: relevant data from an information-rich environment Milbank Q 2010 88 382 403 10.1111/j.1468-0009.2010.00604.x 20860576 PMC3000932

[B23] Canadian Institute for Health Information DAD resource intensity weights and expected length of stay 2005 Ottawa, Ontario

[B24] Centers for Medicare and Medicaid Services ICD-9CM Official guideliens for coding and reporting 2005 Washington DC: United States Department of Health and Human Services

[B25] Averill RF Mullin RL Steinback BA Elai ED Diagnosis Related Groups, Version 9.0 Definition Manual 1991 Washington DC: 3M Health Information System

[B26] Canadian Institute For Health Information CMG + Tool Kit 2007 Ottawa, Ontario

[B27] Elixhauser A Steiner C Harris DR Coffey RM Comorbidity measures for use with administrative data Med Care 1998 36 8 27 10.1097/00005650-199801000-00004 9431328

[B28] Quan H Sundararajan V Halfon P Fong A Burnand B Luthi JC Coding algorithms for defining comorbidities in ICD-9-CM and ICD-10 administrative data Med Care 2005 43 1130 1139 10.1097/01.mlr.0000182534.19832.83 16224307

[B29] Wang CY Baldwin LM Saver BG Dobie SA Green PK Cai Y The contribution of longitudinal comorbidity measurements to survival analysis Med Care 2009 47 813 821 10.1097/MLR.0b013e318197929c 19536031 PMC2701975

[B30] Stukenborg GJ Wagner DP Connors AF Jr Comparison of the performance of two comorbidity measures, with and without information from prior hospitalizations Med Care 2001 39 727 739 10.1097/00005650-200107000-00009 11458137

[B31] Kleinbaum DG Klein M Regression Diagnostics: a self learning text 2002 New York: Springer-Vertag

[B32] Fox J Regression Diagnostics 1991 London: Sage Publications

[B33] Pan W Akaike’s information criterion in generalized estimating equations Biometrics 2001 57 120 125 10.1111/j.0006-341X.2001.00120.x 11252586

[B34] Rabe-Hesketh S Skrondal A Multilevel and longitudinal modeling using strata 2005 College Station: Stata Press

[B35] Begg MD Parides MK Separation of individual-level and cluster-level covariate effects in regression analysis of correlated data Stat Med 2003 22 2591 2602 10.1002/sim.1524 12898546

[B36] Alfandre D Reconsidering Against Medical Advice Discharges: Embracing Patient-Centeredness to Promote High Quality Care and a Renewed Research Agenda J Gen Intern Med 2013 doi:10.1007/S11 606-013-2540-z 10.1007/s11606-013-2540-z PMC3832725 23818160

